# Correction: ‘The deep history of the number words’ (2018), by Pagel and Meade

**DOI:** 10.1098/rstb.2025.0364

**Published:** 2025-11-06

**Authors:** Mark Pagel

**Affiliations:** ^1^ School of Biological Sciences, University of Reading, Reading RG6 6UR, UK

*Phil. Trans. R. Soc. B* **373**, 20160517. (Published online 01 January 2018). (http://dx.doi.org/10.1098/rstb.2016.0517)

There were slight errors in one of the published rates and two of the standard deviations in table 1 of this paper and to the content and rank ordering in table 2 of the Austronesian meanings with the slowest rates of lexical replacement. None of these errors affects any of the statistics or conclusions of the paper.

**Table 1.** Average ± standard deviations of lexical replacement rates for the fundamental vocabulary items and for the low-limit number words, and half lives for the fundamental vocabulary.

replacement rate, per annum	Indo-European (*n* = 200 words)	Bantu (*n* = 102 words)	Austronesian (*n* = 154 words)
overall	0.00020 ± 0.00010	0.00022 ± 0.00009	0.00035 ± 0.00012
*fastest, slowest, ratio (f/s*)	0.0006100, 0.0000047, 130	0.000450, 0.000026, 17	0.00065, 0.000065, 10
*half-life, years: average, shortest, longest,*	3465, 1066, 147 000	3150, 1540, 26 659	1980, 1066, 10 582
‘low-limit’ number words (*one* to *five*) exclude *one*	0.00001 ± 0.00004 (*p* < 0.0001)	0.00011 ± 0.00009 (*p* < 0.003) 0.00006 ± 0.00003 (*p* < 0.00003)	0.00016 ± **0.00012** (*p* < 0.0001) **0.00011** ± **0.00004** (*p* < 0.0001)

**Table 2.** Rank order of rate of lexical replacement for the 11 meanings with the slowest rates of change; rank = 1 is slowest; words ‘one’ to ‘five’ in italics. The probability of all five low-limit number words appearing in the slowest 11 for Indo-European is *p* = 0.0000002; the probabilities of four of the five low-limit number words appearing in the slowest 11 for Bantu is 0.00036 and for Austronesian is 0.00007.

rank	Indo-European (*n* = 200 words)	Bantu (*n* = 102 words)	Austronesian (*n* = 154 words)
1	*two*	eat	*two*
2	*three*	tooth	*three*
3	*five*	*three*	to die
4	who	eye	eye
5	*four*	*five*	*four*
6	I	hunger	fifty
7	*one*	elephant	ten
8	we	*four*	seven
9	when	person	*five*
10	tongue	child	tongue
11	name	*two*	eight

The corrected table 1 is shown here with the corrected values in bold face.

The corrected table 2 is also shown. Only the Austronesian rank ordering is affected. As we originally found, the meanings ‘two’, ‘three’, ‘four’ and ‘five’ still appear among the slowest 11 and so the probability calculations that we reported in the original article still apply. The meanings ‘to pound/beat’ and ‘child’ are no longer among the 11 slowest; the latter’s mention in the Discussion section of the original article no longer applies.

We also point out that the estimated rates for ‘two’, ‘three’, ‘five’ and ‘who’ in Indo-European are numerically equal and so should be considered as tied. This does not affect the probability calculations for the low-limit number words reported in the original text. Their ordering in table 2 reflects our previous finding [1] that number words as a part of speech have slower rates of lexical replacement on average than pronouns.

The word ‘fifty’ has the sixth slowest rate of lexical replacement in Austronesian but it is likely to be a much later borrowing that has been adopted in many languages. For example, it has the fewest number of cognate states in the Austronesian data and by some margin (see see supplementary information to the original article). Excluding ‘fifty’, ‘blood’ becomes the 11th slowest and none of our probability calculations is changed.
